# CAG Somatic Instability in a Huntington Disease Expansion Carrier Presenting with a Progressive Supranuclear Palsy‐like Phenotype

**DOI:** 10.1002/mds.29035

**Published:** 2022-05-05

**Authors:** Ramita Dewan, Zane Jaunmuktane, Monica Emili Garcia‐Segura, Catherine Strand, Edward Wild, Joaquin Villar, Clifton L. Dalgard, Sarah J. Tabrizi, Bryan J. Traynor, Christos Proukakis

**Affiliations:** ^1^ Neuromuscular Disease Research Section, Laboratory of Neurogenetics National Institute on Aging Bethesda Maryland USA; ^2^ Department of Clinical and Movement Neurosciences Queen Square Institute of Neurology, UCL London United Kingdom; ^3^ Queen Square Brain Bank, UCL London United Kingdom; ^4^ Huntington's Disease Centre, Queen Square Institute of Neurology, UCL London United Kingdom; ^5^ Department of Neurodegenerative Disease, Queen Square Institute of Neurology, UCL London United Kingdom; ^6^ Center for Military Precision Medicine Uniformed Services University of the Health Sciences Bethesda Maryland USA; ^7^ Henry M Jackson Foundation for the Advancement of Military Medicine Bethesda Maryland USA; ^8^ Department of Anatomy, Physiology, and Genetics Uniformed Services University of the Health Sciences Bethesda Maryland USA; ^9^ The American Genome Center Uniformed Services University of the Health Sciences Bethesda Maryland USA; ^10^ UK Dementia Research Institute, UCL London United Kingdom

**Keywords:** Huntington's disease; PSP; CAG repeat; mosaicism; TDP‐43; somatic instability

## Author Roles

(1) Research project: A. Conception, B. Organization, C. Execution; (2) Statistical Analysis: A. Design, B. Execution, C. Review and Critique; (3) Manuscript: A. Writing of the First Draft, B. Review and Critique.

R.D.: 1B, 1C, 3B

Z.J.: 1B, 1C, 3B

M.E.G.S.: 1C, 3B

C.S.: 1B

E.W.: 1A, 3B

J.V.: 1C

C.L.D.: 1B,1C, 2B

S.J.T.:1A, 3B

B.J.T.:1A, 1B, 3B

C.P.: 1A, 1B, 3A

## Financial Disclosures

Financial Disclosures of all authors (for the preceding 12 months)

Z.J. received a grant from Aligning Science Against Parkinson's (not related to this work) and belongs to the editorial board member of Acta Neuropathologica. E.W. reports grants from CHDI Foundation, and F. Hoffmann‐La Roche; he receives personal fees from Hoffman La Roche, Triplet Therapeutics, PTC Therapeutics, Annexon Pharmaceuticals, Teitur Trophics, Vico Life Sciences, and Takeda. All honoraria for these consultancies were paid through the offices of UCL Consultants, a wholly owned subsidiary of University College London. University College London Hospitals National Health Service (NHS) Foundation Trust has received funds as compensation for conducting clinical trials for Ionis Pharmaceuticals, Roche, Pfizer, and Teva Pharmaceuticals. S.J.T. received research grant funding from the CHDI Foundation, Vertex Pharmaceuticals, the United Kingdom (UK) Medical Research Council, the Wellcome Trust (ref. 200,181/Z/15/Z), and the UK Dementia Research Institute that receives its funding from DRI, funded by the UK MRC, Alzheimer's Society, and Alzheimer's Research UK. In the past 12 months, through the offices of UCL Consultants, a wholly owned subsidiary of University College London, S.J.T. has undertaken consultancy services for Adrestia Therapeutics, Alnylam Pharmaceuticals, Atalanta Pharmaceuticals, F. Hoffmann‐La Roche, Guidepoint, Locanobio, LoQus23 Therapeutics, Novartis Pharma, PTC Therapeutics, Sanofi, Takeda Pharmaceuticals, Triplet Therapeutics, and University College Irvine. S.J.T. has a patent on the FAN1‐MLH1 interaction and structural analogs for the treatment of disease (application number 2105484.6) licensed to Adrestia Therapeutics. B.J.T. has received funding from Intramural Research Program of the National Institutes of Health, the editorial board member for JAMA Neurology, JNNP, and Neurobiology of Aging. C.P. has received grants from Aligning Science Against Parkinson's and MSA Trust (not related to this work). He belongs to the editorial board member for Frontiers in Neurology (neurogenetics section). R.D., M.E.G.S., J.V., C.D., and C.S. have no disclosures to report.

Pathogenic expansions in huntingtin (*HTT*) may present as progressive supranuclear palsy (PSP)/frontotemporal degeneration, or amyotrophic lateral sclerosis (ALS), without chorea.^1^ We present the first autopsy report of a PSP‐like presentation, with study of somatic CAG expansion. A 68‐year‐old man presented with falls, cognitive impairment, and speech decline over 2 years. He had vertical supranuclear palsy, paucity of speech with a growling character, reduced verbal fluency, apraxia, mild bradykinesia and rigidity, brisk reflexes, but flexor plantars, and no fasciculations. His parents had died relatively young of unrelated causes. The clinical picture resembled PSP, but MRI brain revealed frontoparietal atrophy, with less prominent midbrain and cerebellar atrophy, and small vessel disease. He did not tolerate levodopa or amantadine and rapidly progressed with mobility loss, dysphagia, visual hallucinations, a sweet tooth, and aggression. *C9orf72* testing was negative. After his son developed chorea and was diagnosed with Huntington's disease (HD), he was tested and had 42 CAG repeats, associated with a mean predicted motor onset age of 52.^2^ He died at age 73, without developing chorea. Autopsy revealed typical p62 and 1C2 immunoreactive nuclear inclusions with widespread distribution and severe caudate atrophy (Vonsattel grade 4) and additional limbic TDP43 pathology without motor neuron involvement (Supplementary Fig. S1), in contrast to four ALS‐like cases.^1^, ^3^ Whole genome sequencing of brain DNA revealed no mutations in 111 genes associated with neurodegeneration,^4^ and analysis by ExpansionHunter^5^ confirmed the HD expansion (size: 44 repeats) and normal *C9orf72*.

**FIG. 1:**
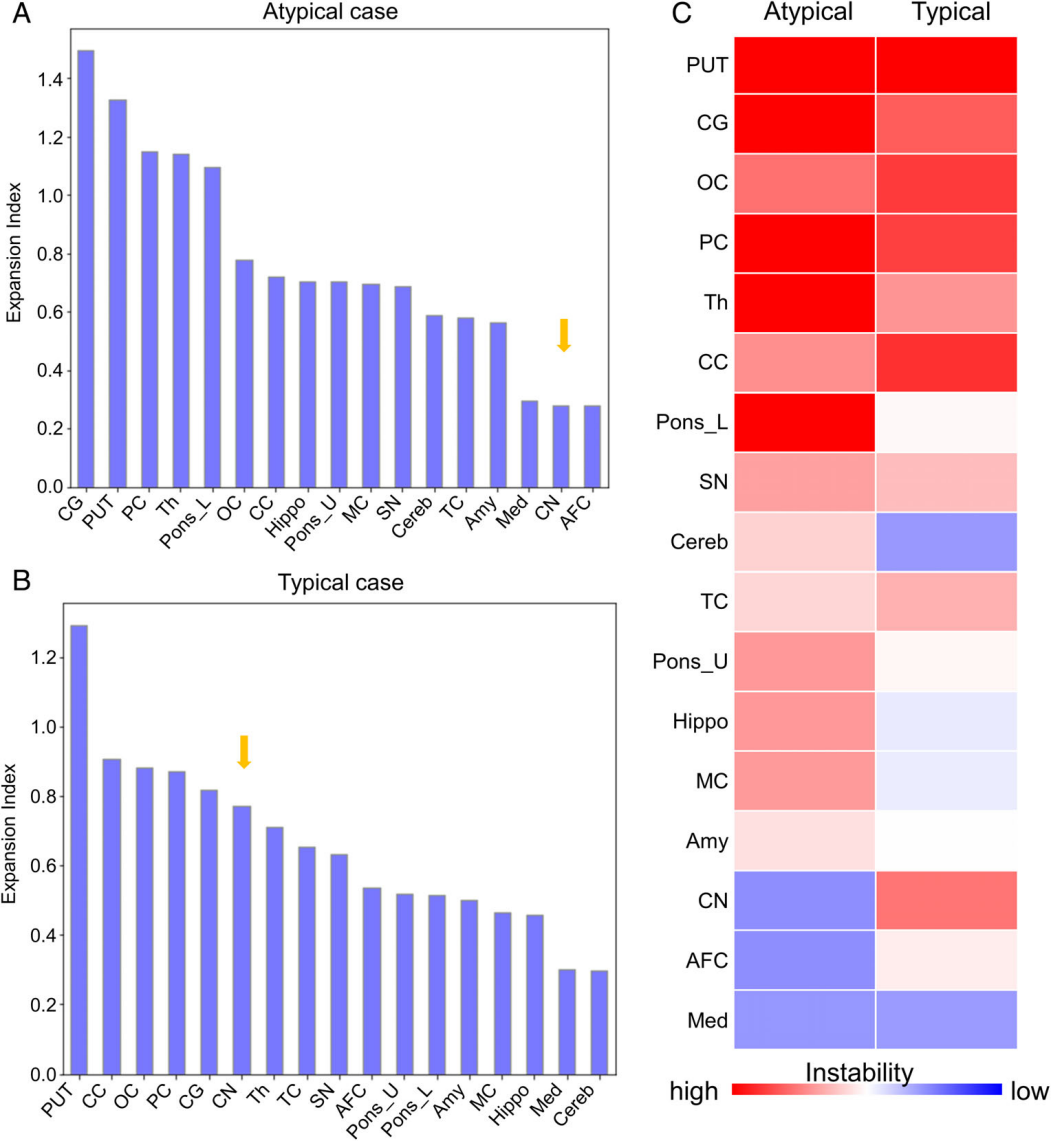
Quantitative analysis of somatic CAG instability in an HTT‐expansion carrier with atypical presentation and a typical HD patient. (**A,B**) Somatic expansion index in each region of (**A**) the atypical case and (**B**) the typical case. The arrow points to the caudate nucleus. (**C**) Heatmap of the expansion index, arranged in order of mean expansion index for each region, from high to low. PUT = putamen, CG = cingulate gyrus, OC = occipital cortex, PC = parietal cortex, Th = thalamus, CC = corpus callosum, Pons_L = lower pons, SN = substantia nigra, Cereb = cerebellum, TC = temporal cortex, Pons_U = upper pons, Hippo = hippocampus, MC = motor cortex, Amy = amygdala, CN = caudate nucleus, AFC = anterior frontal cortex, Med = medulla. [Color figure can be viewed at wileyonlinelibrary.com]

The unusual presentation prompted us to investigate mosaicism because of somatic CAG repeat instability, a well‐known phenomenon,^1^, ^6^, ^7^ in 17 brain regions. In parallel, we analyzed in a blinded fashion another HD male with 42 repeats, typical presentation at 55, and mild caudate atrophy (Vonsattel grade 2). We calculated the somatic expansion index^1^, ^7^ which revealed instability in several regions (Fig. 1), and compared this between them and with published reports. The most striking finding in the atypical case was the relative absence of somatic expansion in the caudate, where it was pronounced in the typical case, consistent with previous reports.^1^, ^6^, ^7^, ^8^ The pontine base showed somatic instability in the atypical patient and was relatively spared in the typical and had also been spared in previously reported typical HD and an ALS case.^1^ The thalamus and amygdala showed instability in the atypical case, less in typical cases, and were relatively spared in an ALS case. The cerebellum was mildly affected in our atypical case, relatively spared in typical cases, and completely spared in ALS.^1^

Although we cannot fully exclude the possibility of a TDP‐43‐related phenotype, rather than atypical HD, the clear HD pathology and limited TDP‐43 pathology support the latter. Our case suggests that a PSP‐like HD presentation may be associated with less detectable somatic CAG instability in the caudate and more in other regions, such as the pons. Because we cannot investigate lost neurons, we cannot determine whether the caudate instability was originally low or appears so because of advanced striatal neuronal loss following somatic expansion.^9^ In the latter case, it may be initial increased somatic CAG expansion that underlies a PSP‐like phenotype. Detailed studies of typical and atypical HD cases are required to determine whether distinct regional somatic instability patterns or co‐existing TDP‐43 pathology influence the phenotype.

## Ethics Statement

All participants gave informed consent before donating brains for research. Ethics approval is provided by the UK National Research Ethics Service (07/MRE09/72). All donors had given informed consent for the use of the brains in research.

## Supporting information


**Fig. S1**. Pathology findings in an HTT‐expansion carrier with atypical clinical presentation. (**A,B**), Atrophy of the caudate nucleus at the level of the nucleus accumbens is evident on macroscopic examination (red arrows). (**C**) At the level of anterior commissure the globus pallidus is reduced in size (blue arrow) and at all levels, dilatation of the lateral ventricle is evident (blue asterisk). (**D,E**) Histology confirms frequent p62 immunoreactive nuclear inclusions in the caudate nucleus (**D**, green arrows), insular cortex, cingulate gyrus and across the cortex of temporal, parietal, and frontal lobes, including the motor cortex (**E**, green arrow), but not within the Betz cells (**E**, orange arrow). The neurons with widespread distribution also show positive diffuse nuclear or dot‐like labelling with 1C2 (shown in the insets of **D** and **E**). (**F**), Cytoplasmic TDP43‐positive neuronal inclusions are frequent in the dentate gyrus (black arrows) and periamygdala cortex, and rare in the amygdala, CA1 hippocampal subregion, subiculum, cortex of the anterior superior frontal gyrus and insula, nucleus accumbens, and caudate nucleus (not shown). (**G**), No TDP43 pathological inclusions are found in the hypoglossal nerve nucleus (orange arrow) or in the primary motor cortex and there is no evidence of corticospinal tract atrophy (not shown). Hyperphosphorylated tau tangle pathology corresponded to Braak and Braak stage II, and there were also occasional argyrophilic grains in the medial temporal lobe and scanty ageing related tau astrogliopathy with thorn‐shaped and fuzzy granular astrocytes in the amygdala. Importantly, there was no evidence of any other primary tauopathy (not shown). Amyloid‐β pathology was restricted to parenchymal deposits in the neocortex and medial temporal lobe (not shown), with the ABC score: A1, B1, C1. Occasional Lewy bodies in the tegmentum of medulla and in the amygdala were also noted (not shown). Scale bar: (**A–C**) 1 cm; (**D,E**) 30 μm; g: 25 μm; insets in **D** and **E**: 15 μm.

## Data Availability

The data that support the findings of this study are available on request from the corresponding author, subject to formal approval by the Queen Square Brain Bank. The data are not publicly available due to privacy or ethical restrictions.
