# Supplementary material for: CAG Somatic Instability in a Huntington Disease Expansion Carrier Presenting with a Progressive Supranuclear Palsy‐like Phenotype
Source: Mov Disord. 2022 May 5;37(7):1555–7. doi: 10.1002/mds.29035 (PMC9308656; doi:10.1002/mds.29035)
Supplement: Supplementary file 1 — Fig. S1. Pathology findings in an HTT‐expansion carrier with atypical clinical presentation. (A,B), Atrophy of the caudate nucleus at the level of the nucleus accumbens is evident on macroscopic examination (red arrows). (C) At the level of anterior commissure the globus pallidus is reduced in size (blue arrow) and at all levels, dilatation of the lateral ventricle is evident (blue asterisk). (D,E) Histology confirms frequent p62 immunoreactive nuclear inclusions in the caudate nucleus (D, green arrows), insular cortex, cingulate gyrus and across the cortex of temporal, parietal, and frontal lobes, including the motor cortex (E, green arrow), but not within the Betz cells (E, orange arrow). The neurons with widespread distribution also show positive diffuse nuclear or dot‐like labelling with 1C2 (shown in the insets of D and E). (F), Cytoplasmic TDP43‐positive neuronal inclusions are frequent in the dentate gyrus (black arrows) and periamygdala cortex, and rare in the amygdala, CA1 hippocampal subregion, subiculum, cortex of the anterior superior frontal gyrus and insula, nucleus accumbens, and caudate nucleus (not shown). (G), No TDP43 pathological inclusions are found in the hypoglossal nerve nucleus (orange arrow) or in the primary motor cortex and there is no evidence of corticospinal tract atrophy (not shown). Hyperphosphorylated tau tangle pathology corresponded to Braak and Braak stage II, and there were also occasional argyrophilic grains in the medial temporal lobe and scanty ageing related tau astrogliopathy with thorn‐shaped and fuzzy granular astrocytes in the amygdala. Importantly, there was no evidence of any other primary tauopathy (not shown). Amyloid‐β pathology was restricted to parenchymal deposits in the neocortex and medial temporal lobe (not shown), with the ABC score: A1, B1, C1. Occasional Lewy bodies in the tegmentum of medulla and in the amygdala were also noted (not shown). Scale bar: (A–C) 1 cm; (D,E) 30 μm; g: 25 μm; insets in D and E: 1 [file MDS-37-1555-s001.docx]

**
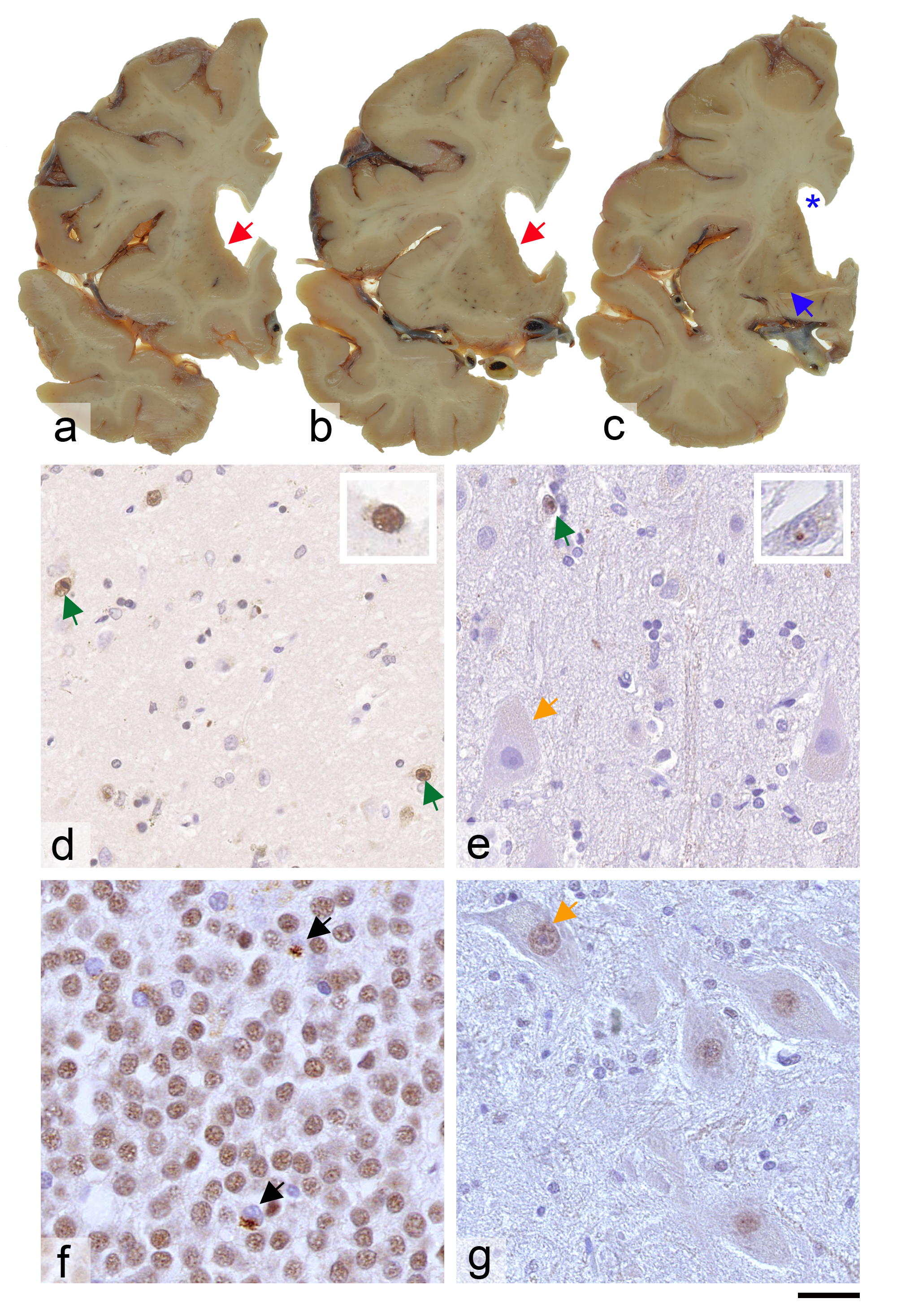
**

**Fig S1 Pathology findings in an HTT-expansion carrier with atypical clinical presentation**

a-b, Atrophy of the caudate nucleus at the level of the nucleus accumbens is evident on macroscopic examination (red arrows). c, At the level of anterior commissure the globus pallidus is reduced in size ( blue arrow) and at all levels, dilatation of the lateral ventricle is evident (blue asterisk). d, e, Histology confirms frequent p62 immunoreactive nuclear inclusions in the caudate nucleus (d, green arrows), insular cortex, cingulate gyrus and across the cortex of temporal, parietal and frontal lobes, including the motor cortex (e, green arrow), but not within the Betz cells (e, orange arrow). The neurons with widespread distribution also show positive diffuse nuclear or dot-like labelling with 1C2 (shown in the insets of d and e). f, Cytoplasmic TDP43-positive neuronal inclusions are frequent in the dentate gyrus (black arrows) and peri-amygdala cortex, and rare in the amygdala, CA1 hippocampal subregion, subiculum, cortex of the anterior superior frontal gyrus and insula, nucleus accumbens and caudate nucleus (not shown). g, No TDP43 pathological inclusions are found in the hypoglossal nerve nucleus (orange arrow) or in the primary motor cortex and there is no evidence of corticospinal tract atrophy (not shown).

Hyperphosphorylated tau tangle pathology corresponded to Braak and Braak stage II, and there were also occasional argyrophilic grains in the medial temporal lobe and scanty ageing related tau astrogliopathy with thorn-shaped and fuzzy granular astrocytes in the amygdala. Importantly, there was no evidence of any other primary tauopathy (not shown). Amyloid-β pathology was restricted to parenchymal deposits in the neocortex and medial temporal lobe (not shown), with the ABC score: A1, B1, C1. Occasional Lewy bodies in the tegmentum of medulla and in the amygdala were also noted (not shown).

Scale bar: a-c: 1cm; d, e: 30µm; g: 25µm; insets in d&e: 15µm.
